# Association of EGFR Exon 19 Deletion and EGFR-TKI Treatment Duration with Frequency of T790M Mutation in EGFR-Mutant Lung Cancer Patients

**DOI:** 10.1038/srep36458

**Published:** 2016-11-04

**Authors:** Norikazu Matsuo, Koichi Azuma, Kazuko Sakai, Satoshi Hattori, Akihiko Kawahara, Hidenobu Ishii, Takaaki Tokito, Takashi Kinoshita, Kazuhiko Yamada, Kazuto Nishio, Tomoaki Hoshino

**Affiliations:** 1 Division of Respirology, Neurology, and Rheumatology, Department of Internal Medicine, Kurume University School of Medicine, Kurume, Fukuoka, Japan; 2 Department of Genome Biology, Kinki University Faculty of Medicine, Osakasayama, Osaka, Japan; 3 Biostatistics Center, Kurume University School of Medicine, Kurume, Fukuoka, Japan; 4 Department of Diagnostic Pathology, Kurume University Hospital, Kurume, Fukuoka, Japan

## Abstract

The most common event responsible for resistance to first- and second-generation (1st and 2nd) epidermal growth factor receptor (EGFR)-tyrosine kinase inhibitor (TKI) is acquisition of T790M mutation. We examined whether T790M is related to clinicopathologic or prognostic factors in patients with relapse of EGFR mutant non-small cell lung cancer (NSCLC) after treatment with 1st or 2nd EGFR-TKIs. We retrospectively reviewed the T790M status and clinical characteristics of 73 patients with advanced or recurrent NSCLC who had been treated with EGFR-TKIs and undergone rebiopsy at Kurume University Hospital between March 2005 and December 2015. T790M mutation was more frequent in patients with *EGFR* exon 19 deletion mutation (63%, 26/41) than in those with L858R mutation (38%, 12/32) (*p* = 0.035). The median total duration of 1st or 2nd EGFR-TKI treatment was significantly longer in patients with T790M mutation than in those without (15.3 months vs 8.1 months, *p* < 0.001). Multivariate analysis revealed that the type of *EGFR* mutation and the total duration of EGFR-TKI treatment were significantly associated with T790M prevalence. Patients with *EGFR* exon 19 deletion mutation who receive long-term EGFR-TKI therapy show a high prevalence of T790M mutation. The present data are potentially important for clinical decision-making in NSCLC patients with *EGFR* mutation.

Prospective clinical trials of first- or second-generation (1st or 2nd) epidermal growth factor receptor (EGFR)-tyrosine kinase inhibitors (TKIs) for treatment of patients with non-small cell lung cancer (NSCLC) harboring *EGFR* mutations have demonstrated remarkable response rates of approximately 70%^1^,^2^,^3^,^4^,^5^,^6^,^7^. Although most NSCLC patients with *EGFR* mutations benefit from treatment with 1st or 2nd EGFR-TKIs, the clinical efficacy differs among individuals, and resistance eventually develops within 9–14 months. Several mechanisms of resistance have been identified, including a second point mutation site where methionine is substituted for threonine at position 790 (T790M) in *EGFR*, amplification of the mesenchymal-to-epithelial transition factor receptor (*MET*), mutation of phosphatidylinositol-4,5-bisphosphate 3-kinase catalytic subunit alpha (*PIK3CA*), transformation to small cell lung cancer, loss of Phosphatase and Tensin Homolog Deleted from Chromosome 10 (*PTEN*), high expression of hepatocyte growth factor, activation of the fatty acid synthase /NF- k B pathway, epithelial-mesenchymal transition, and loss or splice variants of BIM^8^. Of these mechanisms, T790M mutation is the most common and accounts for approximately half of all acquired resistance to 1st or 2nd EGFR-TKIs^9^,^10^.

Recently, third-generation (3rd) EGFR-TKIs, such as Osimertinib and Rociletinib, have been shown to exert a remarkable effect against T790M resistance mutation-positive NSCLC^11^,^12^,^13^. T790M mutation is an important predictive marker for 3rd EGFR-TKIs; therefore, determining the clinicopathologic characteristics of T790M-harboring NSCLC showing relapse after EGFR-TKI therapy is of high clinical relevance. Here we evaluated whether T790M mutation is related to clinicopathologic or prognostic factors in patients with relapse of EGFR-mutant NSCLC after treatment with EGFR-TKIs.

## Results

### Patient characteristics

In the period from March 2005 to December 2015, we identified 193 patients with advanced or recurrent *EGFR*-mutant NSCLC who developed resistance to initial 1st or 2nd EGFR-TKIs treatment. Of these patients, 105 (54%) underwent re-biopsy. Adequate histological specimens were available for 73 of these patients, who were enrolled in the study; 38 (52%) of them had T790M mutation and 35 (48%) did not. All patients were tested for EGFR mutation in pre-EGFR-TKI specimens. Forty-one patients had exon 19 deletion mutation, whereas 32 patients had L858R mutation. Exon 19 deletion and L858R mutation are mutually exclusive (Supplementary Table S1).

At the time of rebiopsy, all patients had been previously treated with gefitinib (n = 58), erlotinib (n = 12), or afatinib (n = 3) as the initial EGFR-TKI. Specifically, 41 (56%), 18 (25%), 12 (16%), and 2 (3%) patients received initial EGFR-TKI as the first, second, third and fourth lines of treatment, respectively. Following relapse after initial EGFR-TKI treatment, 31 patients continued to receive the initial EGFR-TKI treatment at the physician’s discretion.

Table 1 shows data for the association between T790M prevalence and rebiopsy site. There were no significant differences between rebiopsy sites and presence of T790M mtation (*p* = 0.991). Relevant patient characteristics in relation to presence of T790M mutation are summarized in Table 2. T790M mutation was present more frequently in patients with exon 19 deletion mutation (63%, 26 of 41) than in those with L858R mutation (38%, 12 of 32) (*p* = 0.035), in patients with brain metastasis at diagnosis (82%, 14 of 17) than in those without (43%, 24 of 56) (*p* = 0.005), and in patients who received EGFR-TKI treatment for at least 10 months in total (71%, 32 of 45) than in those who did not (21%, 6 of 28) (*p* < 0.001). Other characteristics had no statistical association with the detection of T790M mutation.

### Efficacy of initial EGFR-TKI treatment in relation to presence of T790M mutation

The overall response rate (ORR) and disease control rate (DCR) were significantly higher in the group positive for T790M mutation than in the negative group (ORR: 94.7% vs 60%, *p* = 0.001, respectively; DCR: 97.4% vs 71.4%, *p* = 0.002, respectively).

**Figure 1:**
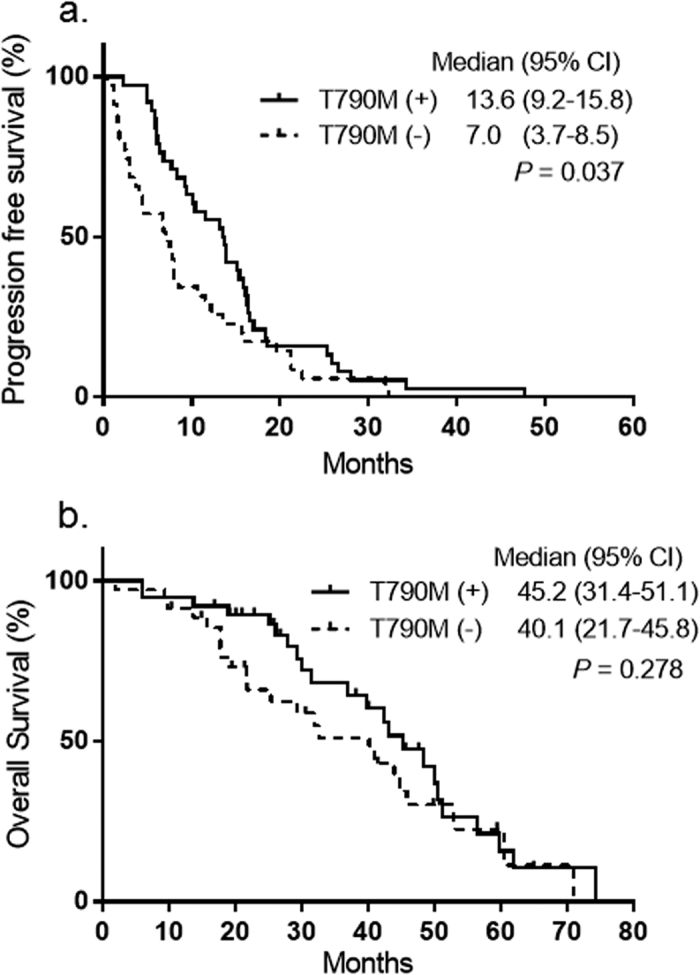
Kaplan-Meier estimates of (**a**) progression-free survival after initial EGFR-TKI therapy and (**b**) overall survival in patients with and without T790M mutation.

The median PFS after initial EGFR-TKI treatment was longer in the T790M mutation-positive group (13.6 months, 95% CI: 9.2–15.8) than in the negative group (7 months, 95% CI: 3.7–8.5) (*p* = 0.037). There was no significant difference in OS between patients with T790M mutation (45.2 months; 95% CI, 31.4–51.1) and those without (40.1 months; 95% CI, 21.7–45.8) (p = 0.278) (Fig. 1). The median total duration of 1st or 2nd EGFR-TKI treatment in patients with T790M mutation was 15.3 months, which was significantly longer than that (8.1 months) in patients without T790M mutation (*p* < 0.001). The last follow-up point was December 2015, and the median follow-up period after initial treatment was 29.7 months.

### Multivariate analysis of T790M mutation prevalence

Multivariate analysis of T790M mutation prevalence was performed on selected variables (Table 3). The type of *EGFR* mutation (exon 19 deletion mutation versus L858R point mutation, *p* = 0.011, OR = 0.21, 95% CI = 0.05–0.71) and total duration of EGFR-TKI treatment (>10 months versus <10 months, *p* < 0.001, OR = 0.09, 95% CI = 0.02–0.28) were significantly associated with the presence of T790M mutation in the multivariate logistic regression models.

## Discussion

Recently, it has become clear that T790M mutation-positive NSCLC shows a high rate of response (approximately 60%) to 3rd EGFR-TKIs^11^,^12^,^13^. Therefore, it is important to determine the characteristics of NSCLCs harboring T790M mutation showing relapse after treatment with 1st or 2nd EGFR-TKIs, as these could have a great impact on treatment strategy. In the present study, we examined the relationship between T790M mutation and clinicopathologic or prognostic factors in patients showing relapse of EGFR-mutant NSCLC after 1st or 2nd EGFR-TKI therapy, and our data showed that a high proportion of patients with EGFR deletion 19 mutation who received long-term 1st or 2nd EGFR-TKI therapy demonstrated a high prevalence of T790M mutation.

Exposure to 1st or 2nd EGFR-TKIs is likely to play an important role in the development of T790M mutation at the time of acquired resistance to 1st or 2nd EGFR-TKIs. In a preclinical study, Chmielecki *et al*. demonstrated that T790M-positive cells were selected after long-term exposure to 1st EGFR-TKIs^14^. Several other studies have shown that T790M-positive cells undergo selection and enrichment during EGFR-TKI treatment^15^,^16^. Kuiper *et al*. reported that the median PFS in a T790M-positive population was 14.2 months, being significantly longer than that (11.1 months) in a T790M-negative population^17^. We also found that PFS after initial EGFR-TKI treatment was significantly higher in the T790M-positive group than in the negative group. These results indicate that long-term exposure to 1st or 2nd EGFR-TKIs might be a predictor of T790M mutation, suggesting that larger studies are warranted to confirm this possibility.

In this study, we observed only the major mutations, i.e. exon 19 deletion and L858R mutation, and found that the T790M positivity rate was significantly higher in patients with exon 19 deletion than in those with L858R mutation. Previous studies have reported similar trends, but no studies except for the study by Nosaki *et al*. have reported a statistically significant association^18^. Unlike our study, the previous studies – except for that by Nosaki *et al*. – observed some minor mutations and included them in their statistical tests for association. Since the frequencies of such minor mutations were small, their inclusion might have led to loss in power in statistical tests. Therefore, we have reanalyzed the four studies^17^,^19^,^20^,^21^ using Fisher’s exact test, focusing on the major mutations, and found that Sun *et al*. and Hata *et al*. also demonstrated a similar trend with statistical significance. Nosaki *et al*. also demonstrated that the T790M mutation was induced more frequently in patients with an exon 19 deletion mutation (55.6%) than in those with an L858R mutation (43.0%) (*P* = 0.05) (Supplementary Table S2). Collectively, T790M mutation was more frequent in patients with *EGFR* exon 19 deletion mutation than in those with L858R mutation.

Recently it was reported that patients with exon 19 deletion mutation showed a better response to 2nd EGFR-TKI than patients with L858R mutation^22^. This difference might be attributable to the biological effects of *EGFR* exon 19 deletion and L858R mutation^23^. It was noteworthy that our study did not demonstrate any significant difference in the median PFS after initial EGFR-TKI therapy and OS between patients with these two types of mutation (Supplementary Fig. S1). The median total duration of 1st or 2nd EGFR-TKI treatment also did not differ significantly between the EGFR exon 19 deletion (13.5 months) and L858R mutation (11.2 months) groups (*p* = 0.367). Therefore, we favor the interpretation that there is no correlation between the type of *EGFR* mutation and the duration of 1st or 2nd EGFR-TKI treatment. Our present results suggest that EGFR exon 19 deletion mutants of NSCLC have a distinct biological phenotype that makes acquisition of T790M mutation more likely upon exposure to 1st or 2nd EGFR-TKIs. However, this hypothesis will require rigorous testing by appropriately designed basic studies.

To analyze the clinical effect of the type of *EGFR* mutation and T790M mutation, we divided the patients into four groups in terms of their type of *EGFR* mutation and positive or negative T790 mutation. The median PFS was 12.5 months in the exon 19 deletion/T790M+ group, 13.8 months in the L858R/T790M+ group, 7.0 months in the exon 19 deletion/T790M- group, and 7.1 months in the L858R/T790M- group (*p* = 0.212). The corresponding median OS values were 43.1, 59.8, 40.9, and 42.4 months, respectively (*p* = 0.465). There was no significant difference in PFS and OS between these groups. It is noteworthy that our patients did not receive any third-generation EGFR-TKIs. Given the high efficacy of third-generation EGFR-TKIs against T790M resistance^11^,^12^,^13^, treatment with 3rd EGFR-TKIs may have contributed to improving the clinical outcomes of patients in who had T790M positivity.

The timing of 1st or 2nd EGFR-TKI withdrawal in NSCLC patients who acquire resistance remains controversial. The IMPRESS study demonstrated no benefit of continued 1st EGFR-TKI with chemotherapy after RECIST progression in comparison to chemotherapy alone^24^. However, in patients with isolated disease progression after development of resistance to 1st EGFR-TKI, continuation of 1st EGFR-TKI in combination with local therapy is feasible, and subsequent treatment can be postponed^25^,^26^,^27^. In our present study, patients with *EGFR* exon 19 deletion mutation who received long-term treatment with 1st or 2nd EGFR-TKI demonstrated a high prevalence of T790M mutation. These results suggesting that continuation of 1st or 2nd EGFR-TKI therapy after RECIST progression seems to be an acceptable option under certain circumstances.

This retrospective study had several limitations. First, the sample size was relatively small. Second, determination of T790M mutation in tumor samples was generally performed using two methods, although there was no significant difference in the incidence of T790M mutation between the methods. Third, the retrospective nature of the study did not allow for a standardized measure of PFS.

In conclusion, we have demonstrated that NSCLC patients with T790M mutation showing disease progression after EGFR-TKI therapy are more likely to have been exposed to EGFR-TKI for a longer period and to harbor EGFR exon 19 deletion mutation. These data could be of potential importance for treatment decision-making after 1st or 2nd EGFR-TKI progression. Further prospective trials and basic research will be needed to confirm our findings.

## Materials and Methods

### Patients

Our study cohort comprised patients with pathologically confirmed advanced (stage IIIB or IV) or recurrent EGFR mutant NSCLC who had been treated with 1st EGFR-TKIs (gefitinib and erlotinib) or 2nd EGFR-TKI (afatinib) and undergone rebiopsy at Kurume University Hospital between March 2005 and December 2015. Using medical records, we retrospectively reviewed the patients’ EGFR T790M mutation status and clinical characteristics. The present study was conducted in accordance with the provisions of the Declaration of Helsinki and was approved by the Institutional Review Board of Kurume University Hospital. Informed consent was obtained from all patients who participated in the study.

### Tumor sampling and T790M mutation analysis

We examined formalin-fixed, paraffin-embedded sections or fresh frozen samples of tumors after acquisition of resistance to EGFR-TKI treatment. Mutational analysis of T790M mutation was performed by the digital polymerase chain reaction (dPCR) using the QX100 Droplet Digital PCR System in accordance with the manufacturer’s instructions (Bio-Rad, Hercules, CA), or the Cobas *EGFR* Mutation Test (Roche Molecular Systems). The primers and probes for T790M mutation were purchased from Bio-Rad. The cutoff value for T790M mutation was set at 3.0 copies on the basis of data from 10 samples of normal DNA.

### Statistical analysis

The associations between T790M mutation and clinical characteristics were analyzed using the chi-squared or Fisher’s exact test. Objective tumor responses were evaluated in accordance with the Response Evaluation Criteria in Solid Tumors (RECIST) 1.1 criteria^28^. Progression-free survival (PFS) was defined as the period from the date of initiation of first-line EGFR-TKI treatment to the date of disease progression or death due to any cause, and overall survival (OS) was measured from initiation of treatment or initial diagnosis until the date of death or last follow-up. The total duration of EGFR-TKI treatment was defined as that between diagnosis and rebiopsy. Survival curves were derived using the Kaplan–Meier method and compared using the log-rank test. Multivariate logistic regression models were prepared to estimate the prevalence of T790M mutation in patients with progression of EGFR-mutant NSCLC after treatment with EGFR-TKIs. All variables with *p* values of < 0.05 were included in the logistic regression models. All tests were two-sided, and differences were considered statistically significant at *p* < 0.05. All of the statistical analyses were conducted using JMP version 11 (SAS Institute Inc., Cary, NC) or Graph Pad Prism version 6.07 for Windows (GraphPad Software, La Jolla, CA; www.graphpad.com).

## Additional Information

**How to cite this article**: Matsuo, N. *et al*. Association of EGFR Exon 19 Deletion and EGFR-TKI Treatment Duration with Frequency of T790M Mutation in EGFR-Mutant Lung Cancer Patients. *Sci. Rep.* **6**, 36458; doi: 10.1038/srep36458 (2016).

**Publisher’s note:** Springer Nature remains neutral with regard to jurisdictional claims in published maps and institutional affiliations.

## Supplementary Material

Supplementary Information

## Figures and Tables

**Table 1:** Details of rebiopsy sites, procedures and T790M mutation prevalence.

Specimen		T790M prevalence	*p*
Lung	37	51% (19/37)	0.987
Pleural effusion	16	44% (7/16)	
Lymph node	5	60% (3/5)	
Cerebrospinal fluid	4	50% (2/4)	
Pericardial effusion	2	50% (1/2)	
Ascites	3	67% (2/3)	
Skin	3	67% (2/3)	
Liver	3	67% (2/3)	

**Table 2:** Patient characteristics and frequency of T790M mutation.

Variables	No	Patients with T790M, No. (%)	*P*
Age, years			
Median	67		
Range	48–82		
Gender			0.782
Male	16	9 (56)	
Female	57	29 (51)	
Histology			NS
Adeno	72	37 (51)	
Squamous	1	1 (100)	
Smoking status			0.408
Never smoker	56	31 (55)	
Smoker	17	7 (41)	
*EGFR* mutation			0.035
Exon 19 deletion	41	26 (63)	
L858R	32	12 (38)	
Stage			0.459
Initially advanced	53	29 (55)	
Recurrent	20	9 (45)	
Rebiopsy sites			0.699
Primary site	33	18 (55)	
Metastasis site	40	20 (50)	
Brain metastasis at diagnosis			0.005
With	17	14 (82)	
Without	56	24 (43)	
Initial EGFR-TKI			0.736
Gefitinib	58	30 (52)	
Erlotinib	12	7 (58)	
Afatinib	3	1 (33)	
Line of initial EGFR-TKI			0.434
First	41	23 (56)	
Second or later	32	15 (47)	
Interval between prior EGFR-TKI and rebiopsy			0.573
<4 m	53	29 (54)	
≧4 m	20	9 (45)	
Immediate prior treatment			0.363
EGFR-TKI	60	33 (55)	
Chemotherapy	13	5 (38)	
Total duration of EGFR-TKI treatment			＜0.001
<10 months	28	6 (21)	
≧10 months	45	32 (71)	
T790M analysis			0.347
Cobas^®^ EGFR Mutation Test	29	13 (45)	
Digital PCR	44	25 (57)	

Abbreviations: EGFR, epidermal growth factor receptor; TKI, tyrosine kinase inhibitor.

**Table 3:** Multivariate analysis of T790M mutation prevalence.

Variable	*P*	OR (95% CI)
Sex (female/male)	0.772	0.84 (0.20–3.37)
*EGFR* mutation (exon 19 deletion/L858R)	0.011	0.21 (0.05–0.71)
Rebiopsy site (primary site/metastasis site)	0.758	0.83 (0.26–2.66)
Total duration of EGFR-TKI treatment (10 mo≤/<10 mo)	<0.001	0.09 (0.02–0.28)
Interval between EGFR-TKI failure and rebiopsy (<4 mo/≥4 mo)	0.170	0.39 (0.08–1.51)

Abbreviations: OR, odds ratio; CI, confidence interval; EGFR, epidermal growth factor; TKI, tyrosine kinase inhibitor; mo, months.
