# Supplementary material for: Association of EGFR Exon 19 Deletion and EGFR-TKI Treatment Duration with Frequency of T790M Mutation in EGFR-Mutant Lung Cancer Patients
Source: Sci Rep. 2016 Nov 4;6:36458. doi: 10.1038/srep36458 (PMC5095551; doi:10.1038/srep36458)
Supplement: Supplementary Information [file srep36458-s1.pdf]

Supplementary Table S1

	**Post-EGFR-TKI T790M**		
**Pre-EGFR-TKI biopsy *EGFR* mutation**	**+**	**-**	**Total**
**Exon 19 deletion**	26	15	41
**L858R**	12	20	32
**Total**	38	35	73

Abbreviations: EGFR, epidermal growth factor receptor; TKI, tyrosine kinase inhibitor.

Supplementary Table S2

Reference	EGFR-TKIs	Pre EGFR-TKI treatment<br>Types of *EGFR* mutation	No	Post EGFR-TKI<br>T790M (%)	*P*
Our study	Gefitinib,	Exon 19 deletion	41	26 (63)	0.035
	Erlotinib or	L858R	32	12 (38)	
	Afatinib				
Nosaki (2016) [18]	Gefitinib,	Exon 19 deletion	162	90 (56)	0.05
	Erlotinib or	L858R	114	49 (43)	
	Afatinib				
Hata (2015) [19]	Gefitinib,	Exon 19 deletion	15	11 (73)	0.048
	Erlotinib,	L858R	24	9 (38)	
	Afatinib				
Kuiper (2014) [17]	Gefitinib,	Exon 19	43	25 (58)	0.342
	Erlotinib,	Exon 21	13	5 (38)	
	Afatinib				
Sun (2013) [20]	Gefitinib or	Exon 19 deletion	31	19 (61)	0.038
	Erlotinib	L858R	18	5 (28)	
Hata (2013) [21]	Gefitinib or	Exon 19 deletion	42	17 (40)	0.217
	Erlotinib	L858R	33	8 (24)	

Abbreviations: EGFR, epidermal growth factor receptor; TKI, tyrosine kinase inhibitor.

Association of EGFR Exon 19 Deletion and EGFR-TKI Treatment Duration with Frequency of T790M Mutation in EGFR-Mutant Lung Cancer Patients

Norikazu Matsuo, MD, Koichi Azuma, MD, PhD, Kazuko Sakai, PhD, Satoshi Hattori, PhD, Akihiko Kawahara, PhD, Hidenobu Ishii, MD, Takaaki Tokito, MD, PhD, Takashi Kinoshita, MD, PhD, Kazuhiko Yamada, MD, PhD, Kazuto Nishio, MD, PhD, and Tomoaki Hoshino, MD, PhD

Supplementary Fig S1

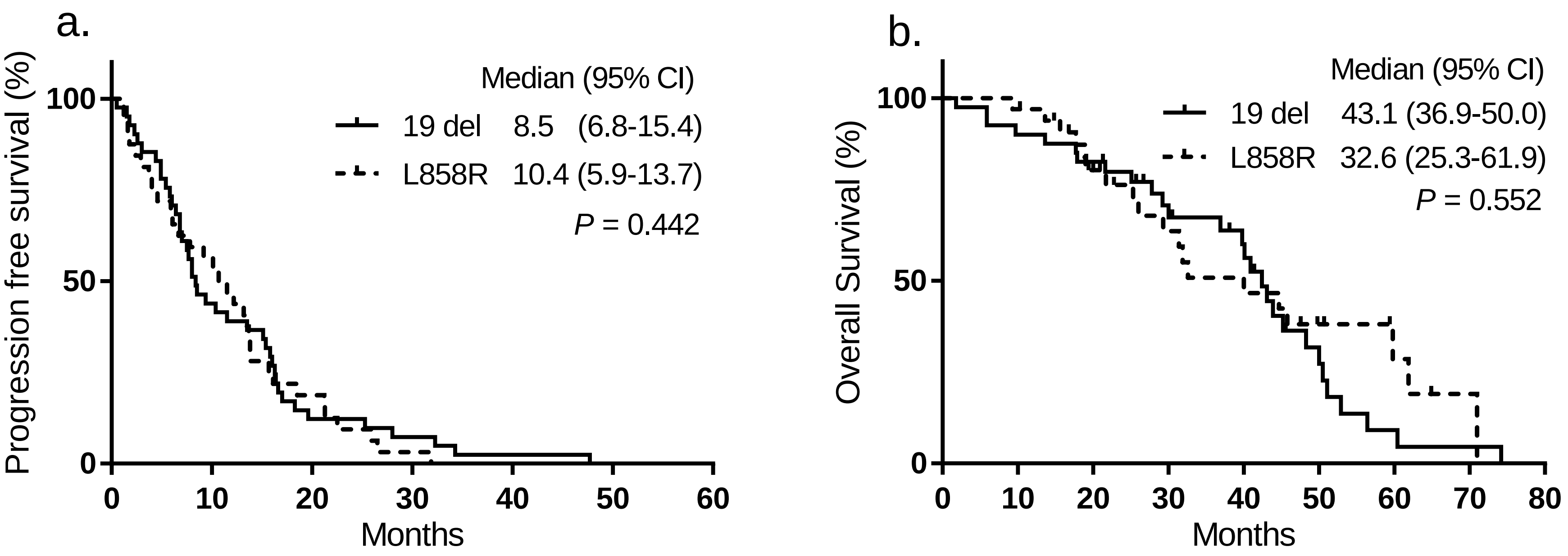

Kaplan-Meier estimates of (a) progression-free survival after initial EGFR-TKI therapy and (b) overall survival in patients with EGFR exon 19 deletion (19 del) and L858R mutation.
